# Latent Cultural Bias in Soundtracks of Western News Coverage From Early COVID-19 Epicenters

**DOI:** 10.3389/fpsyg.2021.686738

**Published:** 2021-07-14

**Authors:** James Deaville, Chantal Lemire

**Affiliations:** ^1^School for Studies in Art and Culture: Music, Carleton University, Ottawa, ON, Canada; ^2^School of Music Arts, London, ON, Canada

**Keywords:** COVID-19, news video, sound analysis, media influence, Wuhan, Tehran, Milan

## Introduction

When news consumers outside China first heard about a new form of pneumonia in Hubei Province in late 2019, it seemed like a distant concern (Hon, 2020). This changed on January 23 with the announcement of lockdown in Wuhan, sparking real concerns for personal well-being should SARS-CoV-2, the virus behind the COVID-19 pandemic, spread (Bansal, 2021). Worldwide interest in the coronavirus spiked, with information flowing from multiple sources including personal interactions, online media, and traditional news media (Lupton and Lewis, 2021). News consumption exponentially increased as a foreign news item became a global health emergency (Hon, 2020). And while researchers in media studies have worked to understand the impact of visual messaging on the global public, surprisingly few address the sonic components of audiovisual COVID-19 reporting.

The sounds of the COVID-19 health crisis, however, are center stage in a growing body of literature in music psychology that investigates the role(s) of music creation and/or consumption in mitigating the impacts of extended lockdown restrictions on personal health and wellness (Chiu, 2020; Fink et al., 2021; Hansen et al., 2021). This research focuses on volitional sounds, i.e., those that people choose to create or hear, and how they function to increase social cohesion under conditions of isolation (MacDonald et al., 2021) or to regulate mood/affect in times of loneliness and ennui (Krause et al., 2021). Yet our lives are filled with aggregates of sounds, known as soundscapes (Schafer, 1969; Truax, 1978; Galloway, 2020), that we passively experience rather than actively select. What about the music, sounds, and silences in our lives that are chosen for us, particularly those that are integral elements of the audiovisual COVID-19 news reporting?

In an ongoing study of the soundtracks from Western news coverage of the pandemic at the first major sites of infection and quarantine—-Wuhan (China), Tehran (Iran), and Milan (Italy)—-we find preliminary evidence that the distribution of sound, silence, and music is regionally and nationally differentiated. These differences could be due to changing schemas associated with the pandemic over time, e.g., as new information becomes available, but as our data shows, early coverage can also reflect cultural biases and potentially promulgate stereotypical and prejudicial representations of race and place. This gives us reason to question the accuracy and fairness of such reporting, as well as to advocate for increased investigations into the sonic elements of COVID-19 reporting as a key area for ongoing pandemic studies. If the sounds of news reports are “the ultimate hidden persuader[s]” (Cook, 2000, p. 128), then they serve as crucial, yet invisible, co-conspirators in the construction of pandemic meanings.

## The Sounds of Pandemic News

### Theory

Studies of pandemic news reporting can draw upon a robust body of literature about prior global infections, most recently about the H1N1 epidemic of 2009–2010 (Duncan, 2009; Angeli, 2012; Vasterman and Ruigrok, 2013). For COVID-19 audiovisual media, researchers have constructed substantial datasets of news items from newspapers (AlAfnan, 2020), social media (Radwan and Radwan, 2020; Tayal and Bharathi, 2021), and television and broadcast media including streaming platforms like Spotify and YouTube (Behluli, 2020; Khatri et al., 2020; Olson et al., 2020). With regard to pandemic music, the Research Topic “Social Convergence in Times of Spatial Distancing” has generated the database CORONAMUSIC (Hansen et al., 2021), which features links to news reports and videos of online music-making and -sharing, and other contributions to the Topic document the benefits of these activities (Mak et al., 2021; Sarasso et al., 2021).

Yet it seems that scholarship has neglected the soundtracks of news reporting, with the ocularcentrism of communication and media studies predominating in analyses of audiovisual pandemic coverage (Parisi et al., 2017; Duff, 2020; Geddes, 2020). Such publications typically do not account for audio elements at play in constructing persuasive news items (Tagg, 1999; Kišiček, 2018; Wang et al., 2021), though knowledge about the unseen sources of manipulation is vitally relevant to a public that believes they are hearing and seeing the truth about the global crisis (Nee and Santana, 2021). The literature of music psychology in particular has addressed how the realm of sound (i.e., vocal tone, contextual sounds, and background music) contributes to the emotional state of the target audience (Tan et al., 2013), which applies in the context of news videos (Pereira et al., 2016; Baumgartner and Wiradhany, 2021).

Taken together, sound studies, musicology, and music psychology provide the researcher with valuable tools for understanding the soundtracks of reportage from the initial hot spots of infection. However, the act of listening to sound and music is never neutral (Eidsheim, 2019). Our perception of the world around us is freighted by cultural values acquired through a lifetime of mediated interpretations (Kassabian, 2013). As Deaville's previous research has established, news music and sound tap into those affective predispositions that are exploited by the media (Deaville, 2006, 2012, 2019). The global news coverage of the pandemic cannot escape the meanings assigned to its soundtracks, which have been produced and consumed within a politics of affect, of “pre-subjective forces and intensities” (Spinks, 2001, 24)—they have corresponded to culturally determined and politically driven biases (Demirtaş-Madran, 2020).

### Materials and Methods

The objects for our preliminary analyses include international news videos from Wuhan, Tehran, and Milan by such networks as CNN, ABC, NBC, and the BBC and by *The Washington Post, The New York Times*, and *The Guardian* (Liseblad, 2019). Data from Google Trends helped to identify the specific dates when news coverage spiked from the early epicenters: January 23–25 for Wuhan (after the initiation of lockdown), February 24–26 for Tehran (after the press conference with the COVID-ill Deputy Health Minister), and March 14 for Milan (the date most densely covered for “balcony music”). A complete list of search terms, sources, dates, video descriptions, and URLs included in our preliminary analyses is available online (Deaville and Lemire, 2021).

In all, preliminary qualitative content analysis considers 60 news reports evenly distributed across the three early hot spots and evaluated according to narrative frame, type of visual content, and type of soundtrack (Ngo et al., 2001). Soundtracks were coded for their use of voiceover; language translation; added musical scoring; non-narrational ambient sounds in the reports, whether performed music, traffic noise, or passing voices (Bello et al., 2018); and uses of silence as a strategic auditory tactic (Le et al., 2019).

Applying the concept of media framing to pandemic newscasting facilitates soundtrack analysis. Framing recognizes how reporters/editors make a cohesive selection of elements from perceived reality to advance a particular interpretation of an event (Entman, 2007). The framer renders that perspective more salient by invoking a “value, theme, stereotype, or symbol that serves to organize and connect topic-relevant information” (D'Angelo, 2017, 1). Media theorists credit frames with possessing the capacity to influence audiences, increasing the emotional appeal and persuasive effect of media messages (Yan et al., 2012; Johannessen, 2015; Uribe, 2020), especially to the extent they are congruent with prevailing cognitive schemas of culture and identity (Wasilewski, 2020).

In studies of news reporting, researchers exploring the psychologies of manipulation and politics have fruitfully applied framing theory (Le Cheminant and Parrish, 2010; Wood et al., 2018) and schema theory (Wood et al., 2018; Wojdynski and Evans, 2020). As Mustafa argues, “associated schemata are joined together to produce frame-systems, which facilitate an efficient decoding of what is observed” (2020, p. 130). As applied to the sonic elements of news reporting, framing theory reveals curated soundscapes that carry media messaging as readily as the visual and content-based communication of the news item (Facchinetti, 2021). And granted that these non-narrational sounds and silences are largely unnoticed by the public, they can become prime vehicles for covertly (mis-)leading audiences to adopt certain attitudes toward the news (Baum, 2002). Identifying the relevant cognitive schemata has involved investigating the role of sound in construction of national identity, conceptions of national ideology and politics, and cultural and societal values (Abbas, 2020; Mutua and Oloo Ong'ong'a, 2020).

## Three Early Epicenters

### Wuhan

This study suggests that sound—or more accurately, the relative absence of sound—was complicit in the Western media's construction of the Chinese response to COVID-19 through the lockdown on January 23. The audiovisual news reports of Western correspondents featured certain common themes: the Chinese state's absolute control in quarantining its citizens and Wuhan's lockdown as a sonic and visual “ghost city” [see the sources in Deaville and Lemire (2021)].

While criticizing China's lockdown for its severity and harshness (McGregor, 2020), the foreign press presented Wuhan as an incapacitated city, marked by silent, empty streets (D'Amore, 2020). The sights and sounds of quarantine were constructed by the eyes and ears of the Western press, which largely excluded signs of life from inside containment. The direct recipients of the virus' debilitating effects, human bodies, were hardly in evidence in the earliest video coverage from the metropolis under lockdown. These representations correspond to Western pre-dispositions toward the Chinese state as oppressive and its citizens as “invisible” (Lee, 2004, p. 80)—one could say that the reporters colonized the racialized and “disappeared” Wuhanese and deprived them of physical appearance and voice.

### Tehran

One particular moment during the pandemic's early days struck the media as particularly news- and sound-worthy for correspondents' reports from Tehran: the press conference on February 24. There, Iranian Deputy Health Minister Iraj Harirchi is heard coughing and seen sweating, to be diagnosed only hours afterward as having COVID-19. The Western press seemed to magnify the sound and sight of his distress, as if to represent the “chaos” of the theocracy in Tehran.

This type of mediated bias is not unusual for news representations of Iran (Hargreaves and Staetsky, 2019), especially in the wake of the pandemic (Devi, 2020; Murphy et al., 2020). The surveyed Western news videos problematize any semblance of official control over the pandemic in Tehran, featuring unabated street sounds, untranslated bureaucrat speeches, and repurposed social media videos of Iranian doctors dancing, to suggest the ineffectiveness of government orders (for an especially noteworthy example, see CBC News, The National). One cannot rule out the possibility that the bases for such images and sounds reside in what some commentators designate as “racialized Islamophobia,” rooted in Orientalism and Colonialism (Kirtsoglou and Tsimouris, 2018; Grace and Heins, 2021).

### Milan

Music figured prominently in international news reports from Italy in mid-March, whilst the coronavirus spread throughout the country and challenged the public under lockdown to connect and relieve the “quarantedium,” as documented and studied elsewhere in this research topic (Chiu, 2020; Granot et al., 2021). The solution in Milan and other urban centers was so-called “balcony music,” whereby apartment dwellers sang or performed on various instruments from their balcony, at times alone and at times in chorus with their neighbors. These extra-ordinary examples of music-making caught the ears of the press, which disseminated the footage to news consumers in North America and Europe (Facchin, 2020; Tejedor et al., 2020).

The apparently impromptu balcony performances fulfilled a Western sonic bias toward the response to the health crisis in the “land of song” (Bennett, 1896; Ketterson, 2015). Visually present and sonically resonant, these musicians—amateur and professional—model socially-cohering corona-musicking behavior in the face of the incapacitating health disaster. Here musical sounds fill the media frames, invoking race, ethnicity, and nationality to connect with favorable audience dispositions.

## Discussion

As Hansen et al. (2021) and Eden et al. (2020) demonstrate, music can serve as a powerful agent for personal well-being and social cohesion in stressful times *when selected and/or curated for those purposes*. However, the sonic domain also engages the public in non-volitional contexts, such as the broadcast and online news that become crucial sources of information in crisis conditions. We hope to have illustrated how the unnoticed music, sounds, and silences of news soundtracks can reflect cultural biases and potentially influence their consumers.

Nevertheless, the preliminary findings are based on a relatively small dataset. Augmenting the data sources would be desirable to obtain a more robust confirmation of results. That would involve consulting a larger number of audiovisual items from a greater spread of professional news providers, as provided in the media sub-corpus from the CORONAMUSIC DATABASE (Hansen et al., 2021) as well as in other relevant news corpora like Coronavirus Corpus (2020-) and NOW Corpus (News on the Web, 2010).

Moreover, our sources include only news reports generated by and for the Anglophone world, which does not account for other national perspectives and soundtracks (Deaville and Lemire, 2021). Expansion of the research would necessarily include videos by major European news networks, to ensure that the biases uncovered do not only characterize press reactions from North America and the United Kingdom (Le and Phi, 2021). At later stages of research, the project should consider soundscapes in reporting from other parts of Europe, the Middle East, and Asia.

Data from news video clips could be compiled into a database that allows for parametric categorization (e.g., by date, network, narrative frame, type of visual content, style and amount of soundtrack). Then they could undergo quantitative analysis, to determine the comparative prevalence of voices, sounds, music, and silences in soundtracks as sorted by location. It would also be possible to use the database for quantitative assessment of bias, according to procedures developed for analyzing news videos by cultural sociology (Al Ibrahim and Shi, 2020; Moscadelli et al., 2020) and social psychology (Paluck et al., 2017; Chien, 2019).

## Author Contributions

JD designed and conducted the study, analysed the data, and created a first draft of the manuscript. CL contributed to the presentation of data analysis, the development of a narrative for final presentation and revisions to the manuscript. Both authors contributed to the article and approved the submitted version.

## Conflict of Interest

The authors declare that the research was conducted in the absence of any commercial or financial relationships that could be construed as a potential conflict of interest.
